# Noise Reduction Techniques and Scaling Effects towards Photon Counting CMOS Image Sensors

**DOI:** 10.3390/s16040514

**Published:** 2016-04-09

**Authors:** Assim Boukhayma, Arnaud Peizerat, Christian Enz

**Affiliations:** 1 Integrated Circuits Lab (ICLAB), École Polytechnique Fédérale de Lausanne (EPFL), Microcity, Rue de la Maladière 71, Neuchâtel 2000, Switzerland; christian.enz@epfl.ch; 2 Laboratoire de l’ Électronique et Technologies de l’ Information (Leti), Commissariat a l’ Énergie Atomique (CEA), Rue des Marthyrs 17, Grenoble 38000, France; arnaud.peizerat@cea.fr

**Keywords:** CMOS, image sensors, temporal read noise, 1/*f* noise, thermal noise, correlated multiple sampling, deep sub-electron noise

## Abstract

This paper presents an overview of the read noise in CMOS image sensors (CISs) based on four-transistors (4T) pixels, column-level amplification and correlated multiple sampling. Starting from the input-referred noise analytical formula, process level optimizations, device choices and circuit techniques at the pixel and column level of the readout chain are derived and discussed. The noise reduction techniques that can be implemented at the column and pixel level are verified by transient noise simulations, measurement and results from recently-published low noise CIS. We show how recently-reported process refinement, leading to the reduction of the sense node capacitance, can be combined with an optimal in-pixel source follower design to reach a sub-$0.3\;\;e_{rms}^{-}$ read noise at room temperature. This paper also discusses the impact of technology scaling on the CIS read noise. It shows how designers can take advantage of scaling and how the Metal-Oxide-Semiconductor (MOS) transistor gate leakage tunneling current appears as a challenging limitation. For this purpose, both simulation results of the gate leakage current and 1/f noise data reported from different foundries and technology nodes are used.

## 1. Introduction

The idea of an image sensor with photon counting capability is becoming a subject of interest for new applications and imaging paradigms [1,2,3]. Such a device must have an input-referred read noise negligible compared to a single electron. Among the state-of-the-art imaging devices, single photon detectors may appear to be the best candidate for such an application [4]. Historically, micro-electronics could not provide readout chains with noise levels as low as deep sub-electron. Hence, the solution was to introduce a gain at the level of the photon-electron conversion. In photomultipliers tubes (PMTs) and single photon avalanche photodiodes (SPADs), the electron generated by the incident photon is accelerated and multiplied to a number of electrons from a few hundred in PMTs to millions in SPADs. Such a signal level can be easily detected and quantized into two logic levels, since the number of incident photons during the period of detection is assumed to be much less than one. However, these devices present the following disadvantages [5]. First, they are limited to the case of single photon detection. In other words, the arrival of one photon and multiple photons are not distinguished. Second, these devices suffer from a dead time and after pulse following each photon detection, blinding the device for a certain time. The third limitation is related to the low resolution and fill factors of focal plane arrays using such devices. Additionally, they use high voltages, which are not compliant with standard CMOS image sensor (CIS) processes.

During the last decade, CISs have seen their performance increasing remarkably in terms of dynamic range, speed, resolution and power consumption. With a lower cost and better on-chip integration, CISs replaced progressively the charge coupled devices (CCDs) in many applications and enlarged the market of electronic imaging devices. In terms of sensitivity, the quantum efficiency has been improved to reach levels as high as 0.95 [3]. The fill factors have been constantly improved. The dark current in the pinned photodiodes (PPDs) has been reduced to levels making the process of electron-hole pair generation noiseless for integration times around tens of ms. The read noise has also been dramatically reduced to reach deep sub-electron levels [6,7,8]. Hence, CIS technologies are advanced enough to envisage the photon counting possibility.

Besides the quantum efficiency, this paper discusses the possibility of performing photon counting, with standard CIS, essentially from the read noise perspective. Starting from the analytical expressions of the input-referred noise, the noise reduction mechanisms at the circuit, device and process level are discussed and verified with simulation, measurements and data reported in recent works. The impact of the combination of different techniques is also analyzed, and the noise levels that can be reached with state-of-the-art technology in standard processes are quantified. This paper also shows how the technology downscaling can be used to reduce the read noise and how the gate leakage current could limit this advantage.

## 2. CMOS Image Sensors and Photon Counting Requirements

**Figure 1:**
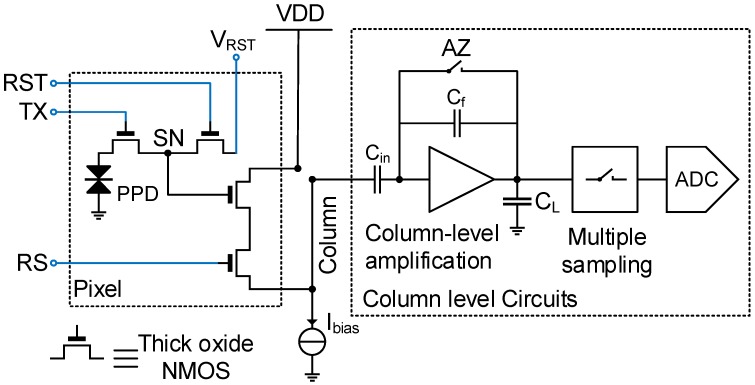
Schematic of a conventional low noise CMOS image sensor (CIS) readout chain. RST, reset switch; TX, transfer gate; RS, row selection switch; SN, sense node; PPD, pinned photodiode; AZ, auto-zero; VDD, supply voltage .

**Figure 2:**
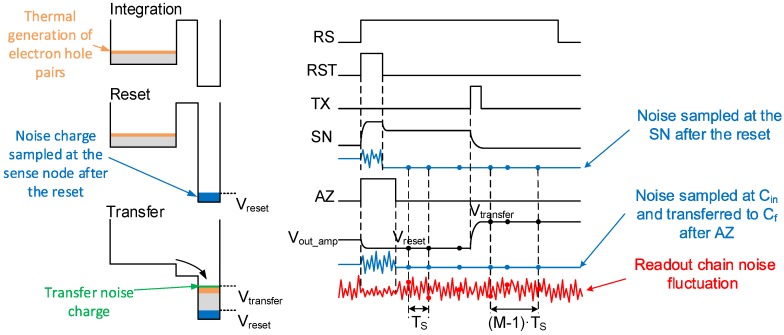
Timing diagram of the conventional CIS readout chain of Figure 1 with noise mechanisms affecting the signal at the PPD and the readout chain levels.

Figure 1 shows the schematic of a conventional low noise CIS readout chain. The corresponding timing diagram is shown in Figure 2. It also shows the potential profile across the PPD, the transfer gate (TX) and the sense node (SN) during the three phases of operation: the integration, the reset and the transfer phases. During the integration time, the PPD accumulates the electrons generated by the incident photons. During the readout, the pixel is connected to the column through the row selection switch (RS), then the reset switch (RST) is closed in order to set the SN voltage higher than the pinning voltage of the PPD. The voltage level at the SN after the reset is read with the in-pixel source follower (SF) and sampled at the end of the readout chain. The potential barrier between the PPD and the SN is controlled by the transfer gate (TX). When the barrier is lowered, the charges accumulated in the PPD are transferred to the SN. The SN voltage level after the transfer is sampled at the output of the readout chain. The reset and transfer samples are then differentiated. This operation is called correlated double sampling (CDS) [9].

Figure 2 depicts also the different noise sources affecting the signal in the CIS apart from the photon shot noise. During the integration, the charge originating from the thermal generation of electron-hole pairs in the depleted region of the PPD (the dark current) can corrupt the signal. In state-of-the-art CIS, the dark current in PPDs has been reduced to a few e${}^{-}/$s. Hence, for exposure times below hundreds of ms, the dark current can be neglected.

The reset of the SN leaves a kT/C noise charge held at the SN. This noise is as high as several electrons in the case of a SN capacitance of a few fF. However, for 4T pixels, it is canceled thanks to the CDS readout scheme, as depicted in the timing diagram of Figure 2.

The charge transfer from the PPD to the SN can be affected by the noise related to the charge deficit due to incomplete transfer and lag [10,11]. Unlike the sampled reset kTC noise, this noise is not canceled by the CDS. The charge transfer noise has been extensively studied for CCDs [12,13] because an efficient charge transfer is crucial in such devices. In state-of-the-art CIS with 4T pixels, values of the lag as low as 0.1% have been reported. Thus, the lag can be neglected compared to the read noise in the low light context. The transient noise related to the lag is believed to behave as a shot noise [11], similarly to buried channel CCDs [13]. However, with a lag below 1%, this noise can be neglected in low light conditions. It is also believed that trapping mechanisms in the silicon oxide interface under the transfer gate also contribute to the transfer non-idealities [10,14,15,16], giving rise to a Random Telegraph Signal (RTS)-like noise.

Finally, the readout of the SN reset and transfer voltages is affected by random fluctuations due to the readout chain noise; starting with the in-pixel SF and noise coupling of the TX and RST lines with the SN, the power supply noise and ending with the column-level circuitry and analog-to-digital converters (ADCs). The column-level amplification is introduced in order to minimize the contribution of the next circuit blocks to the input-referred total noise, e.g., buffers, sample-and-holds and ADC. The column-level amplifier also limits the bandwidth in order to minimize the thermal noise [8]. A switched capacitor amplifier is usually used. An auto-zero (AZ) is performed in order to reset its feedback capacitor and to reduce its offset and 1/f noise [9]. When the AZ switch is opened, the noise is sampled at the integration capacitor and transferred to the output. This sampled noise is also canceled thanks to the CDS. Low noise CIS readout chains may also include correlated multiple sampling (CMS) that can be implemented with analog circuitry [17,18] or performed after the ADC [19]. CMS consists of averaging *M* samples after the reset and *M* other samples after the transfer with a sampling period $T_{S}$, then calculating the difference between the two averages.

**Figure 3:**
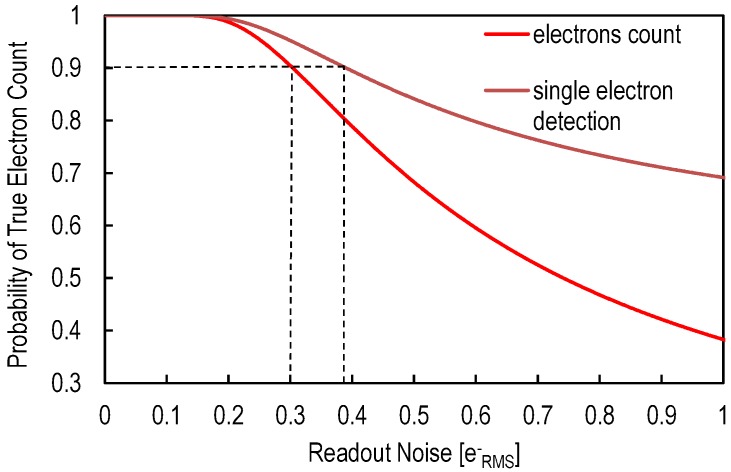
Probability of a true photo-electron count and single photo-electron detection as a function of the input-referred readout noise.

With a careful design, the readout noise originating from the pixel and column-level amplifier is the dominant noise source in CIS. Figure 3 shows the calculated probability of a true photo-electron count and a single photo-electron detection as a function of the input-referred readout chain noise by assuming a Gaussian distribution of noise and using the error function. Based on Figure 3, 90% accuracy requires a read noise below $0.4\;e_{rms}^{-}$ for single photo-electron detection and $0.3\;e_{rms}^{-}$ for photo-electron count. Recently reported works are today closer than ever to these limits [7,8,20]. A detailed noise analysis of the readout noise is therefore necessary in order to determine the key design and process parameters that can be used for further noise reduction.

## 3. Read Noise in CIS

**Figure 4:**
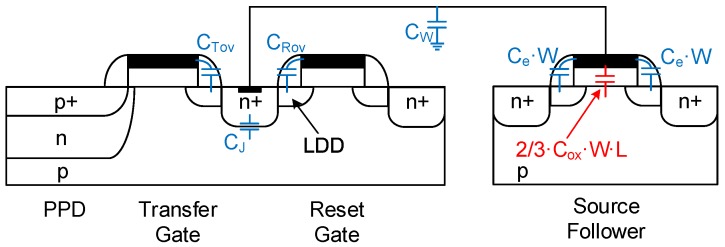
Cross-section of a conventional 4T pixel showing the different parasitic elements contributing to the sense node capacitance.

In a conventional CIS readout chain, three readout noise sources can be distinguished: thermal noise, 1/f noise and leakage current shot noise. For each noise source, the variance at the output of the readout chain is first calculated and then referred to the input as a noise charge. Hence, the pixel conversion gain is a key parameter in the noise analysis. The pixel conversion gain can be calculated using a small-signal analysis of the pixel. It is crucial to take into account the effect of parasitic capacitances. Figure 4 presents a schematic of a 4T pixel section view showing all of the parasitic capacitances connected to the sense node. These include the overlap capacitances of the transfer and reset gates, $C_{Tov}$ and $C_{Rov}$, respectively, the sense node junction capacitance, $C_{J}$, and the parasitic capacitance related to the metal wires, $C_{W}$. These capacitances are independent of the in-pixel SF. Their sum is defined as:

(1) $$C_{P}=C_{Tov}+C_{Rov}+C_{J}+C_{W}$$

**Figure 5:**
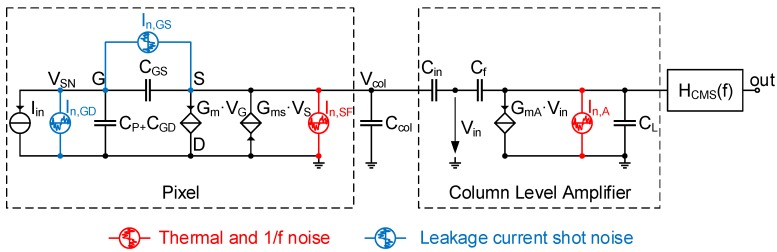
Small-signal analysis of the CIS readout chain depicted in Figure 1 showing the different readout noise sources considered in the analysis.

Figure 5 presents a simplified small-signal schematic of the CIS readout chain of Figure 1. This small-signal schematic is used to calculate the conversion gain together with the noise and signal transfer functions. Based on the detailed analytical calculation presented in [8], the conversion gain of a conventional CIS 4T pixel can be expressed as:

(2) $$A_{CG}=\frac{\frac{1}{n}}{C_{P}+C_{e}\cdot W+\left(1-\frac{1}{n}\right)\left(C_{e}\cdot W+\frac{2}{3}C_{ox}\cdot W\cdot L\right)}$$

Here, *n* is the slope factor of the in-pixel SF [21] defined as $G_{ms}/G_{m}$, where $G_{m}$ and $G_{ms}$ are the SF gate and source transconductances, respectively. $C_{e}$ is the extrinsic capacitance per unit width of the in-pixel source follower transistor. It includes the overlap and fringing capacitances as depicted in Figure 4. $C_{ox}$ is the SF oxide capacitance per unit area.

### 3.1. 1/f Noise

Under the long-channel approximation, the gate-referred 1/f noise power spectral density (PSD) of a MOS transistor operating in the saturation region is commonly expressed as:

(3) $$S_{V_{g}}\left(f\right)=\frac{K_{F}}{C_{ox}^{2}\cdot W\cdot L\cdot f}$$

Here, *W* and *L* are the gate width and length; $C_{ox}$ is the oxide capacitance per unit area; and $K_{F}$ is a 1/f noise process and bias-dependent parameter. This empirical model is easy to use for hand calculation and remains valid even for advanced CMOS technologies for adequate gate widths and lengths [22]. The parameter $K_{F}$ can be expressed as [21,23]:

(4) $$K_{F}=K_{G}\cdot k\cdot T\cdot q^{2}\cdot \lambda \cdot N_{t}$$

where *k* is the Boltzmann constant, *T* is the absolute temperature, *q* is the electron charge, *λ* is the tunneling attenuation distance (≃0.1 nm) [24], $N_{t}$ is the oxide trap density and $K_{G}$ is a bias-dependent parameter. It has been shown in [21] that $K_{G}$ is close to unity when the transistor is operating in the weak and moderate inversion regime.

Most analog circuit simulators use the Berkeley Short-channel Model (BSIM) to predict the 1/f noise behavior of circuits. It is important to establish a relationship between the parameters used by the simulator and the simple equation used for hand calculations in order to best exploit the noise calculation results. The oxide trap density is the key process-dependent parameter. In the BSIM model, it is referred to as the noise parameter A (noiA) [25].

**Figure 6:**
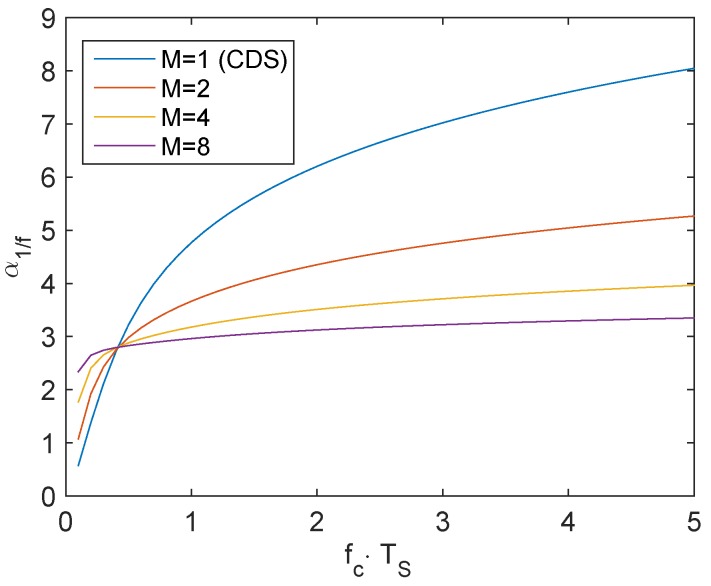
Numerical calculation of the parameter $\alpha_{1/f}$ from Equation (6) as a function of the ratio between the cutoff frequency of the readout chain and the sampling frequency of the correlated sampling T $f_{c}\cdot T_{S}$ for a simple CDS and CMS with different orders *M*.

It is well known that the 1/f noise PSD is inversely proportional to the gate area. In low noise CIS readout chains, the transistors located outside the pixels array can be designed with gate dimensions much larger than the in-pixel source follower transistor. In this case, the latter becomes the dominant 1/f noise source in the readout chain, and the other 1/f noise sources can be neglected. Based on the small-signal schematic of Figure 5 and the calculation detailed in [8], the input-referred 1/f noise can be expressed as:

(5) $$\bar{Q_{1/f}^{2}}=\alpha_{1/f}\cdot \frac{K_{F}{\left(C_{P}+2C_{e}\cdot W+\frac{2}{3}C_{ox}\cdot W\cdot L\right)}^{2}}{C_{ox}^{2}\cdot W\cdot L}$$

where $\alpha_{1/f}$ is a unitless circuit design parameter reflecting the impact of the CMS noise reduction on the 1/f noise. Based on the detailed analytical calculation [26], it can be expressed as:

(6) $$\alpha_{1/f}=\int_{0}^{\infty }\frac{1}{f}\cdot \frac{4}{M^{2}}\frac{sin^{4}\left(\pi \cdot M\cdot T_{S}\cdot f\right)}{sin^{2}\left(\pi \cdot T_{S}\cdot f\right)}\cdot \frac{1}{1+{\left(\frac{f}{f_{c}}\right)}^{2}}df$$

where $f_{c}$ is the cutoff frequency of the column-level amplifier, which is assumed to be lower than the SF stage bandwidth. $T_{S}$ is the sampling period of the correlated sampling. $\alpha_{1/f}$ is calculated numerically and plotted as a function of $f_{c}.T_{S}$ in Figure 6. It shows that $\alpha_{1/f}$ is weekly dependent on $T_{S}$ when *M* is higher than two. In this case, $\alpha_{1/f}$ ranges between three and four.

### 3.2. Thermal Noise

The thermal noise of a MOS transistor operating in saturation is modeled by a drain current source that adds to the signal. The drain current noise PSD is commonly expressed as [21]:

(7) $$S_{I_{d}}\left(f\right)=4\cdot k\cdot T\cdot \gamma \cdot g_{m}$$

where $g_{m}$ is the gate transconductance of the transistor and *γ* is the excess noise factor given by $\frac{2n}{3}$, for a long-channel transistor biased in strong inversion [21].

In a conventional CIS readout chain, besides the power supply and bias voltage noise, there are two dominant thermal noise sources: the in-pixel SF transistor operating in saturation and the column-level amplifier. The latter makes the noise sources from the next stages (ADC, CMS, *etc*.) negligible when enough gain is provided. The two dominant noise sources are uncorrelated; thus, their noise PSDs add. We assume that the bandwidth of the in-pixel SF stage is limited by the column-level amplifier. We consider that the column-level gain is provided by a closed-loop operational transconductance amplifier (OTA). Using the small-signal analysis of the SF stage and the column-level amplifier [8], the thermal noise voltage variance at the output of the column-level amplifier is calculated. It is then referred to the input using the column-level and conversion gain Equation (2), resulting in:

(8) $$\bar{Q_{th}^{2}}=\alpha_{th}\cdot \frac{kT}{A_{col}\cdot C}\left(\frac{\gamma_{SF}G_{m,A}{\left(\frac{2}{3}C_{ox}\cdot W\cdot L+2C_{e}\cdot W+C_{P}\right)}^{2}}{G_{m,SF}}+\frac{\gamma_{A}}{A_{CG}^{2}}\right)$$

where $C=C_{L}+\frac{C_{in}}{A_{col}+1}$. Here, $C_{L}$ and $C_{in}$ are the integration and load capacitances of the column-level amplifier. $\gamma_{SF}$ and $\gamma_{A}$ are the noise excess factors corresponding to the in-pixel source follower transistor and the OTA of the column-level amplifier, respectively. $G_{m,SF}$ and $G_{m,A}$ are the transconductances of the in-pixel SF stage and column-level OTA, respectively. $\alpha_{th}$ is a unitless circuit design parameter dependent on the circuit or processing techniques used after the column-level amplification stage. In the case of CMS, $\alpha_{th}$ is given by [26]:

(9) $$\alpha_{th}=\frac{1}{\pi f_{c}}\int_{0}^{\infty }\frac{4}{M^{2}}\frac{sin^{4}\left(\pi \cdot M\cdot T_{S}\cdot f\right)}{sin^{2}\left(\pi \cdot T_{S}\cdot f\right)}\cdot \frac{1}{1+{\left(\frac{f}{f_{c}}\right)}^{2}}df≃\frac{2}{M}$$

Note that for proper settling of the signal between sampling instants, $2\pi \cdot f_{c}\cdot T_{S}$ has to be typically larger than five, and under such conditions, $\alpha_{th}$ can simply be approximated by $\frac{2}{M}$.

### 3.3. Leakage Current Shot Noise

During the readout, the charge transferred to the SN may be corrupted by all of the leakage currents through the junctions and gate oxide due to tunneling. Since these leakage currents are due to barrier control processes, they give rise to shot noise. As shown in the small-signal schematic of Figure 5, the leakage current shot noise can be modeled by two noise current sources: $I_{n,GD}$ and $I_{n,GS}$. $I_{n,GD}$ represents the shot noise of all of the leakage currents flowing between the SN and the ground, which includes the SN junction leakage and the SF gate oxide tunneling current that sinks into the bulk and the drain. $I_{n,GS}$ represents the shot noise associated with part of the SF gate oxide tunneling current that flows to the source. The unilateral PSD of the current shot noise can be expressed as [27]:

(10) $$S_{I_{L}}\left(f\right)=2\cdot q\cdot I_{L}$$

where $I_{L}$ is the mean value (DC current) of the total leakage current. It can be shown that both shot noise components $I_{n,GD}$ and $I_{n,GS}$ have the same transfer function magnitude, between the noise current source and the output of the column level amplifier. The leakage current shot noise PSD at the output of the column level amplifier can therefore be simplified as:

(11) $$S_{L,Amp}\left(f\right)=S_{I_{L}}\left(f\right)\cdot \frac{A_{CG}^{2}\cdot A_{col}^{2}}{{\left(2\pi f\right)}^{2}}\cdot \frac{1}{1+{\left(\frac{f}{f_{c}}\right)}^{2}}$$

Note that $I_{L}$ is the sum of all of the sense node leakage currents. The noise PSD after the CMS, taking into account the impact of aliasing, can be expressed as:

(12) $$S_{L,CMS}\left(f\right)=sinc{\left(\pi fT_{S}\right)}^{2}\cdot \frac{4}{M^{2}}\frac{sin^{4}\left(\pi \cdot M\cdot T_{S}\cdot f\right)}{sin^{2}\left(\pi \cdot T_{S}\cdot f\right)}\cdot \sum_{n=-\infty }^{+\infty }S_{L,Amp}\left(f-nT_{S}\right)$$

**Figure 7:**
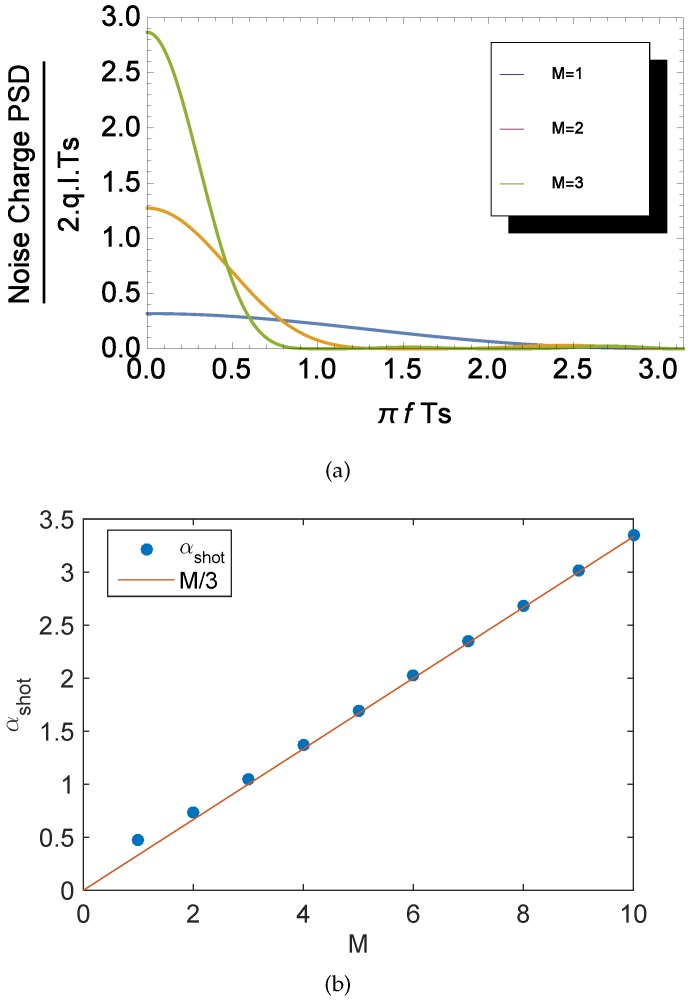
Input-referred shot noise PSD (**a**) and variance (**b**), normalized to $2\cdot q\cdot I_{L}\cdot T_{S}$ as a function of the correlated multiple sampling (CMS) order *M*.

Figure 7a shows a plot of the input-referred shot noise PSD, normalized to $2\cdot q\cdot I_{L}\cdot T_{S}$. It can be noticed that due to the $1/f^{2}$ term in Equation (11), the PSD is independent of $f_{c}$, and the area of the PSD increases with *M*. It can be shown that the input-referred charge variance due to the total leakage current’s shot noise can be expressed as:

(13) $$\bar{Q_{L}^{2}}=2\cdot \alpha_{shot}\cdot q\cdot I_{L}\cdot T_{S}$$

with:

(14) $$\alpha_{shot}=\int_{0}^{\infty }\frac{1}{\pi }\cdot \frac{sin{\left(M\cdot x\right)}^{4}}{{\left(x\cdot sin\left(x\right)\right)}^{2}}\cdot \frac{1}{1+{\left(\frac{x}{f_{c}\cdot T_{S}}\right)}^{2}}dx≃\frac{M}{3}\;\left(for\;M\geq 2\right)$$

Note that the shot noise current sources feature a white PSD. However, when integrated in the SN capacitance, they give rise to a Wiener process [28]. The variance of this noise is thus expected to rise with the readout time. In order to evaluate the impact of the CMS on the leakage current shot noise, $\alpha_{shot}$ is calculated numerically and plotted in Figure 7b as a function of *M*. In the case of a simple CDS, $\alpha_{shot}$ is equal to 0.5; hence, the shot noise variance is given by $q\cdot I_{L}\cdot T_{S}$, which corresponds to a typical case of a Wiener process [28]. Figure 7b also shows that, in the general case, the leakage current shot noise increases linearly with $T_{S}\cdot M$.

## 4. CIS Read Noise Reduction Techniques

### 4.1. Column-Level Techniques

**Figure 8:**
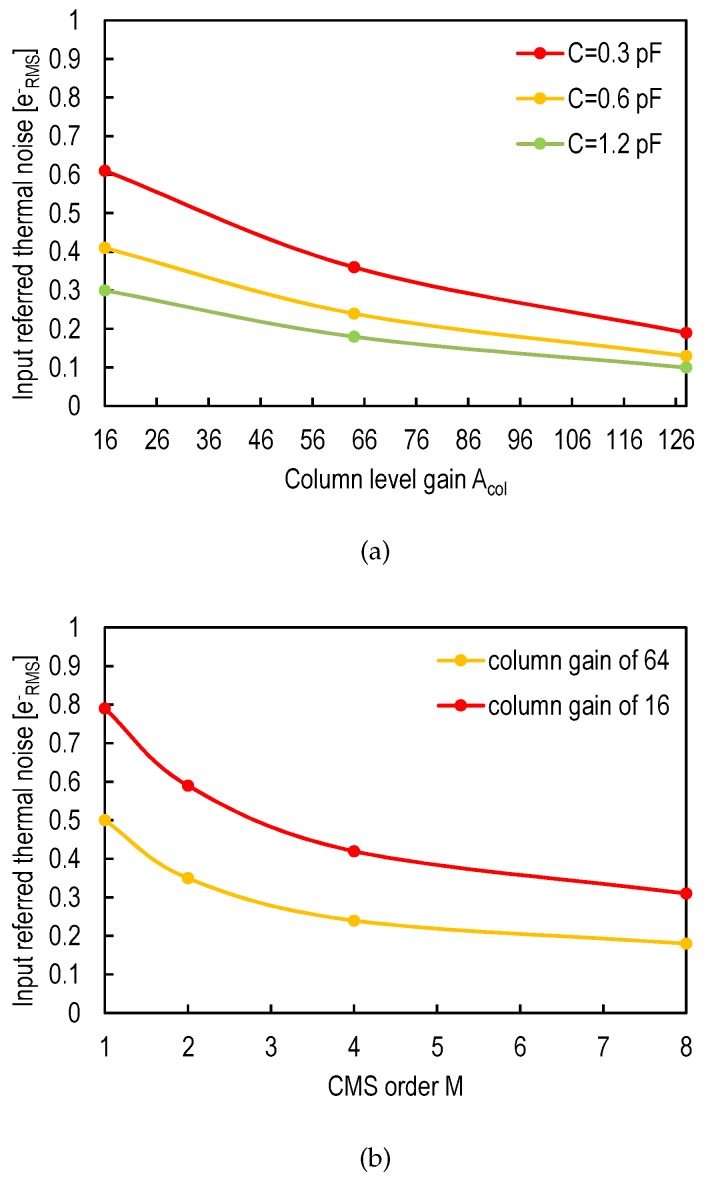
Input-referred thermal noise, obtained from transient noise simulations, of a CIS readout chain, with a 4T pixel (standard NMOS source follower) with a conversion gain of 85μV$/e^{-}$, column amplification (closed loop gain with the operational transconductance amplifier (OTA)) and CMS implemented with the analog circuit presented in [17], as a function of: (**a**) the column-level gain $A_{col}$ for different values of *C* and a simple CDS (M = 1); (**b**) the CMS order *M* for different values of the column-level gain $A_{col}$ and C=0.2 pF.

Based on Equation (8), the parameters that can be used to reduce the readout thermal noise independently of the pixel design are the column-level gain $A_{col}$, the capacitance *C* acting on the bandwidth of the column-level amplifier and the CMS order *M* that determines the value of the parameter $\alpha_{th}$. For thermal noise, the column-level gain $A_{col}$, the capacitance *C* and the CMS order *M* all have the same impact on the input-referred noise. In order to validate this result, transient noise simulations [29] have been performed on a conventional CIS readout chain with a 4T pixel using a standard thick oxide NMOS source follower transistor, a column-level amplifier based on an OTA with a feedback capacitance $C_{f}$ and the passive CMS circuit presented in [17]. Figure 8a shows the impact of the column-level gain and bandwidth control on the input-referred noise when a simple correlated double sampling is used after the column-level amplifier. Figure 8b shows the impact of the correlated multiple sampling on the input-referred thermal noise for different column-level gains. These simulation results show that the thermal noise can be reduced drastically using only the column-level parameters.

**Figure 9:**
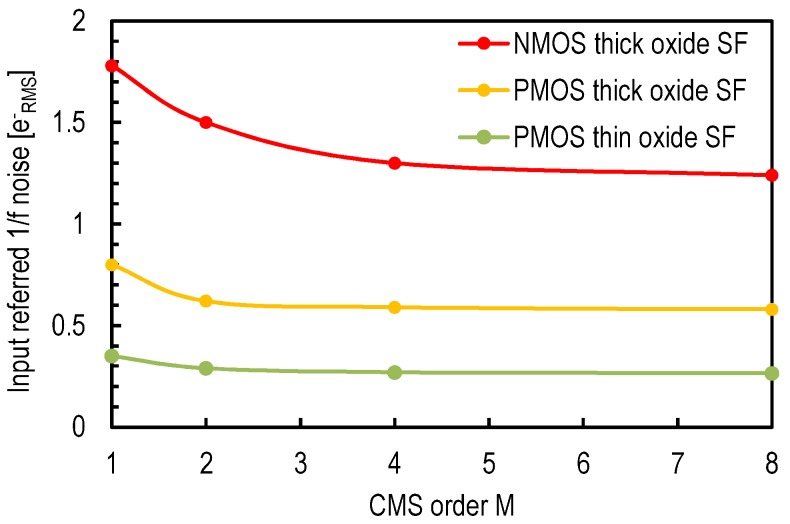
Input-referred 1/f noise of a CIS readout chain, with 4T pixel, column amplification and CMS [17], obtained with transient noise simulations, as a function of the CMS order *M* for different in-pixel source follower transistor types. The pixels with thick oxide NMOS and PMOS SFs feature a conversion gain of about 85μV$/e^{-}$, while the thin oxide SF based pixel features a conversion gain of 185μV$/e^{-}$.

Based on Equation (5), $\alpha_{1/f}$ is the only parameter in the input-referred 1/f noise expression that is independent of the pixel-level device and process parameters. As shown in Section 3.1, $\alpha_{1/f}$ decreases with the CMS order. Figure 9 shows transient noise simulations of the input-referred 1/f noise for readout chains with different in-pixel SF types as a function of the CMS order. Figure 9 demonstrates that even if the CMS comes with some 1/f noise reduction, the impact of the device parameters (SF type) is much more significant.

### 4.2. Pixel-Level Techniques

The thermal noise can be reduced to extremely low levels by implementing column-level circuit techniques as shown in Figure 8a,b. Consequently, further reduction of the thermal noise at the pixel level is less efficient, and noise optimization at the pixel level should be mostly focused on reducing the remaining and dominant 1/f noise. Equation (5) is the starting point for the 1/f noise optimization and suggests different approaches, including proper device selection, design optimizations, as well as process improvements.

#### 4.2.1. Reduce the Capacitance $C_{P}$ and the Source Follower Transistor Overlap Capacitance

**Figure 10:**
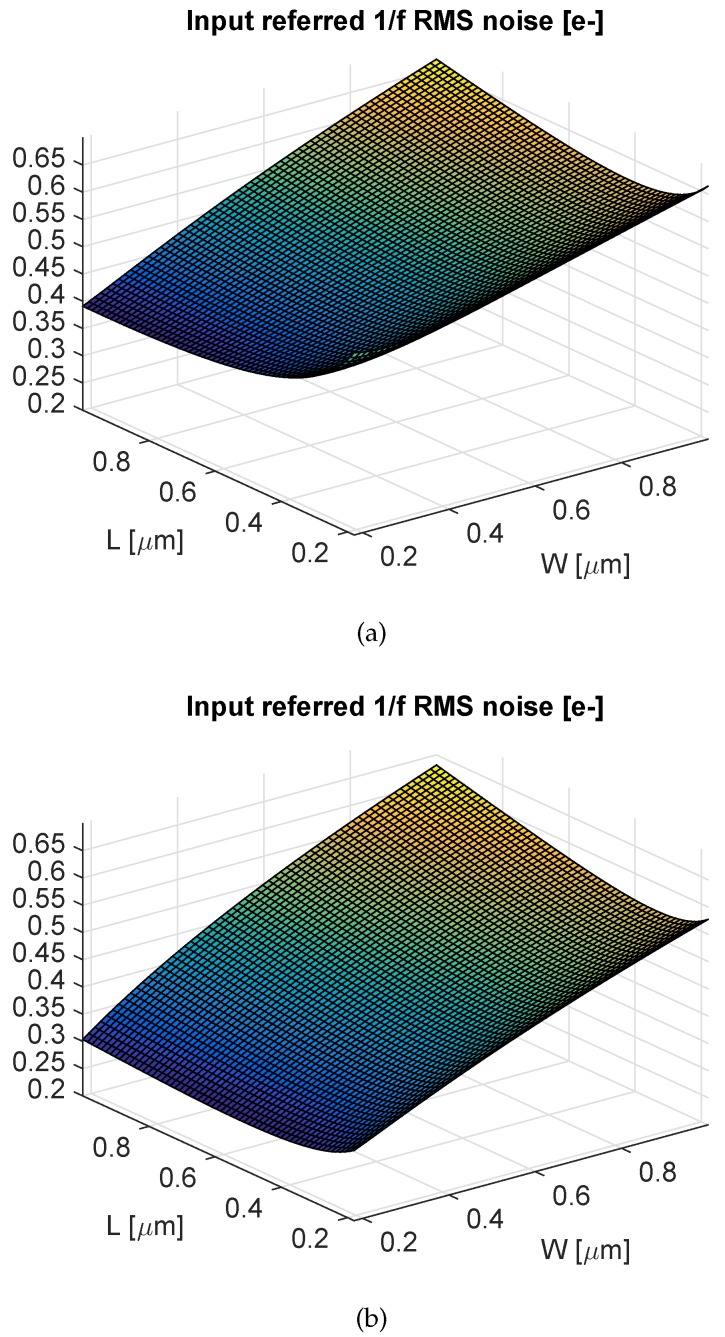
The calculated input-referred 1/f noise, based on Equation (5), as a function of the in-pixel source follower width W and length L for a thin oxide transistor in a 180-nm technology where $C_{gse}=C_{gde}=0.95$ fF/*μ*m, $C_{ox}=9.5$ fF/*μ*m${}^{2}$, $K=10^{-11}\;$F${}^{2}$V${}^{2}$/m${}^{2}$, $\alpha_{CMS}=3$, and in (**a**) $C_{P}=0.75$ fF, it corresponds to the SN capacitance in a standard process, (**b**) $C_{P}=0.25$ fF corresponds to an SN capacitance reduced with process-level optimization.

This point remains an active research topic. Careful layout is not enough to significantly decrease the contributions of the wiring parasitic capacitances, the transfer and reset overlap capacitances, as well as the junction capacitance of the floating diffusion. For this purpose, process improvements are necessary. Many recent works presenting sub-electron readout noise CIS actually focused on this point. In [30], different process-level techniques have been presented leading to the reduction of $C_{P}$. It has been shown that the omission of the low doped drains (LDDs) used in standard CMOS transistors reduces effectively the gate overlap capacitances. Furthermore, increasing the depletion depth under the floating diffusion by reducing the doping concentration reduces the junction capacitance. The combination of these techniques led to a $C_{P}$ reduction of about 47%. In [7], the capacitance $C_{P}$ has been reduced by using an idea called “virtual phase”, well known in CCDs, consisting of creating a potential profile that isolates the floating diffusion from the transfer gate. In this way, the overlap capacitance between the transfer gate and the floating diffusion (denoted $C_{Tov}$ in Figure 4) is dramatically reduced. Furthermore, the channel width of the reset transistor is reduced by controlling the doping profile in order to reduce the overlap between the reset gate and the floating diffusion (denoted $C_{Rov}$ in Figure 4). However, this was obtained at the cost of a low pixel full-well capacity and a relatively higher lag. In [20], $C_{Tov}$ is reduced by introducing a special implant isolating the transfer gate from the SN, and $C_{Rov}$ is reduced by omitting the reset transistor. However, this requires the reset to be performed with a high voltage clock of 25 V connected directly to an implant close to the SN. Figure 10 shows the impact of the $C_{P}$ reduction through a plot of the calculated input-referred 1/f noise as a function of the gate width and length, based on Equation (5), for a $C_{P}$ of 0.75 fF, corresponding to a standard process, and a $C_{P}$ of 0.25 fF, corresponding to the one that could be obtained through advanced process refinements [30]. Figure 10 shows the effectiveness of this $C_{p}$ reduction, which leads to a reduction of the input-referred 1/f noise from 0.4 to $0.3\;e_{rms}^{-}$.

#### 4.2.2. Reduce the 1/f Noise Process Parameter $K_{F}$ (the Oxide Trap Density $N_{t}$)

**Figure 11:**
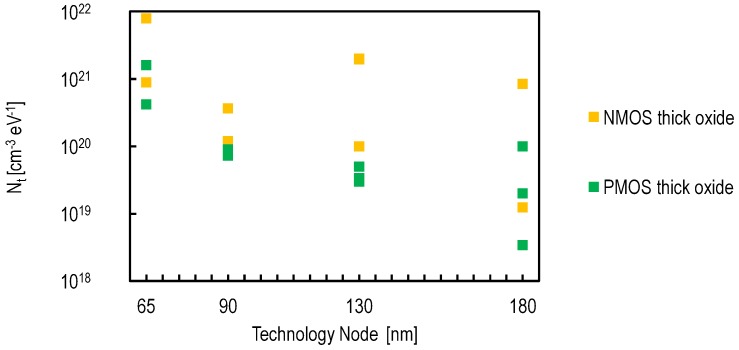
The oxide trap density $N_{t}$, of PMOS and NMOS thick oxide transistors, as a function of the technology node based on measurement results reported in design kits from different foundries.

This point can be addressed through design choices and technological improvements. It is known that buried channel devices have a lower 1/f noise by featuring a lower $K_{F}$ parameter. This is likely due to the fact that the charge carriers are kept away from the silicon oxide interface [31]. It has been shown that using buried channel NMOS source followers leads to sub-electron noise performance [19]. From a design aspect, thick-oxide transistors that operate at voltages as high as 3.3 V are commonly used in CIS pixels. Figure 11 shows the oxide trap density of PMOS and NMOS thick oxide transistors from different foundries and technology nodes. It shows that PMOS transistors feature, generally, a $K_{F}$ parameter lower than NMOS transistors. Using an in-pixel PMOS source follower transistor also led to a sub-electron noise performance [32]. The drawback of in-pixel PMOS transistor is the reduction of the fill factor due to the spacings imposed by the layout design rules and the possible quantum efficiency reduction if the PMOS n-well is too close to the PPD.

#### 4.2.3. Increase the Oxide Capacitance per Unit Area $C_{ox}$

Based on Equation (5), the 1/f noise can also be reduced by increasing the oxide capacitance per unit area of the in-pixel SF. From a design perspective, this corresponds to the selection of a thin oxide SF instead of the traditional thick oxide transistor. In most CIS processes, all of the gates included in the pixel feature thick oxides, since the transfer gate and the reset gate are controlled by high voltages (3.3V); the SF is also chosen as a thick oxide transistor to exploit a high dynamic range. In [8], it has been shown how a thin oxide transistor can be implemented in a CIS pixel without degrading the dynamic range or dramatically reducing the fill factor at the benefit of a much reduced input-referred 1/f noise.

#### 4.2.4. Use a Minimum Gate Width and an Optimal Length

Based on Equation (5), it can be shown analytically [33,34] or numerically using the a plot of Equation (5) *versus* the gate width and length that the lowest input-referred 1/f noise corresponds to the minimum gate width and an optimal length generally slightly higher [33]. Figure 10 illustrates this principle on a practical example. It shows a plot of the input-referred 1/f noise as a function of the gate width and length for a pixel based on a thin oxide PMOS SF of a 180-nm CMOS process for two different values of $C_{P}$. It shows how, for both $C_{P}$ values, the input-referred 1/f noise can be reduced by choosing a minimum SF gate width and a slightly larger length. Note that the reduction of the SF gate size might increase the probability of RTS noise occurrence [35]. However, the amplitude of the RTS noise is inversely proportional to the gate area [36]; thus, it would be also reduced by using a minimum SF gate width.

#### 4.2.5. Thin Oxide Source Follower: A Good Match

**Figure 12:**
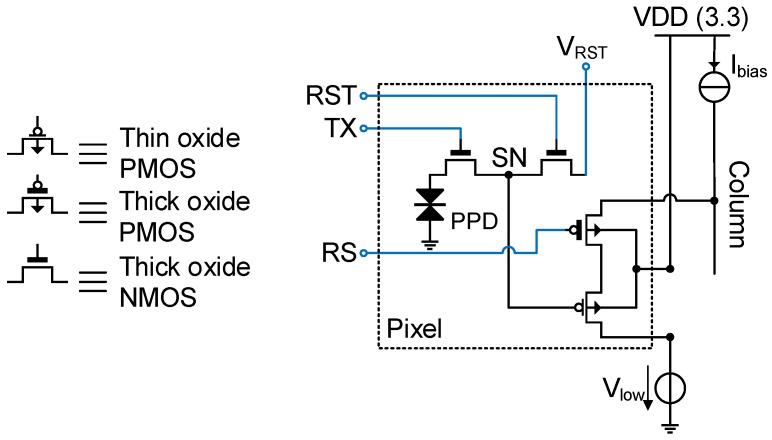
Schematic of the recently-proposed pixel [8] based on a thin oxide PMOS source follower.

From the designer’s perspective, the points mentioned in Section 4.2.3 and Section 4.2.4 can both be addressed by using a thin oxide SF. Indeed, A thin oxide SF with a minimum gate width features also lower overlap capacitances. It is important to verify that the choice of a thin oxide transistor does not come at the cost of a negative impact on the 1/f noise process parameter $K_{F}$. A thinner oxide is expected to come with a better control of the gate over the channel and, therefore, a lower $K_{F}$. The oxide trap density of thin oxide PMOS transistors and thick oxide NMOS transistors of different foundries and technology nodes has been compared in [8] based on data reported in design kits. This comparison showed that the thin oxide PMOS transistors generally feature a lower oxide trap density. Thus, using a thin oxide PMOS source follower addresses the points of Section 4.2.2, Section 4.2.3 and Section 4.2.4 at once. Figure 12 shows the schematic of a 4T pixel based on a thin oxide PMOS SF. In order to validate this idea, a transient noise simulation is performed on three readout chains based respectively on a standard thick oxide NMOS, a thick oxide PMOS and a thin oxide PMOS source follower in a 180-nm CIS process. The compared readout chains share the same column level amplification and CMS circuit presented in [17]. Figure 9 shows the impact of the in-pixel source follower transistor type, as well as the CMS order *M* on the input-referred 1/f noise. The PMOS SF-based readout chain features a lower 1/f noise than the NMOS-based one thanks to the increase of $C_{ox}$ and the reduction of the parameter $K_{F}$. The readout chain based on the thin oxide PMOS SF features the lowest input-referred noise because its SF cumulates a lower $K_{F}$, a higher oxide capacitance per unit area and a lower minimum width.

It is important to verify that these techniques are not harmful in terms of the thermal noise. Equation (8) shows that the thermal noise is reduced by increasing the conversion gain. Thus, using a thin oxide SF with smaller gate dimensions is also expected to reduce the input-referred thermal noise.

**Figure 13:**
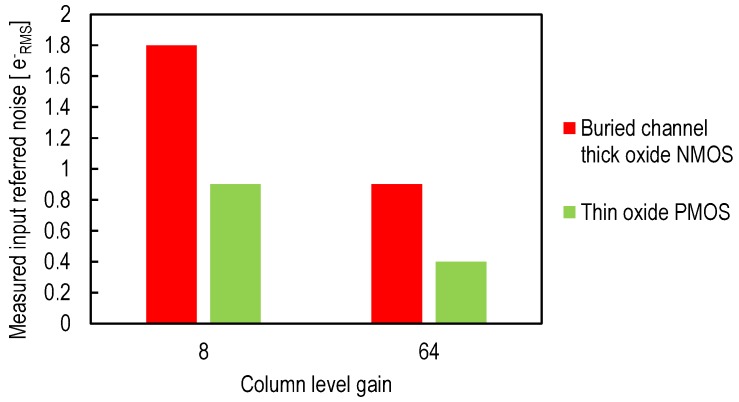
The measured input-referred total noise of a CIS readout chain, with 4T pixel and column amplification, for two column level gains $A_{col}$, for an in-pixel buried channel thick oxide NMOS source follower and a thin oxide PMOS source follower from [8].

The benefits of using a thin oxide SF transistor have been confirmed with measurement results. A test chip comparing pixels based on thin oxide PMOS source followers with state-of-the-art pixels with thick oxide buried channel NMOS source followers has been presented in [8]. Figure 13 shows the measured average total input-referred noise of the two different pixels for two column-level gain values and with a simple CDS. Figure 13 shows how both thermal (at low column gain) and 1/f noise (at high column gain) are dramatically reduced thanks to the implementation of the source follower with a thin oxide PMOS transistor.

**Figure 14:**
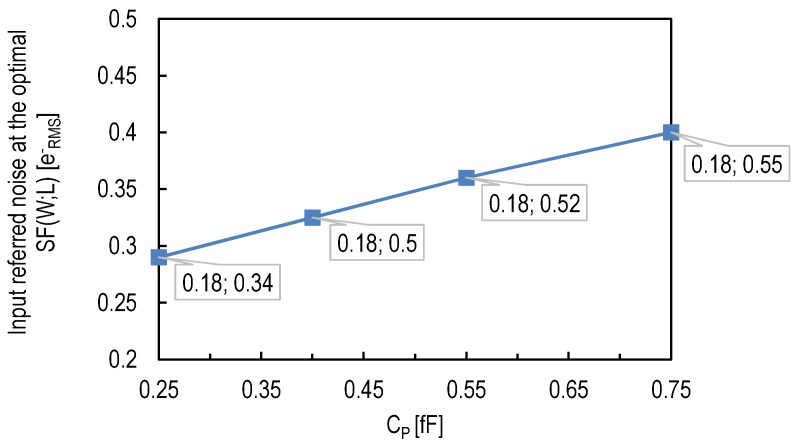
The input-referred 1/f noise as a function of the capacitance $C_{P}$ determined by the floating diffusion, wiring and parasitic capacitances independent from the source follower transistor. $C_{P}=\;0.75$ fF corresponds to the case of a conventional sense node junction, transfer gate and transistors. $C_{P}=0.55$ fF corresponds to the case of transfer and reset gates without low doped drains [30]. $C_{P}=0.4$ fF corresponds to the case of transfer and reset gates without low doped drains and a sense node without channel stop underneath [30]. $C_{P}=0.25$ fF corresponds to the case of more advanced process refinements as [7,30]. The numbers in labels correspond to the minimum width and optimum length as discussed in Section 4.2.4.

The tested pixel was designed using a standard CIS process and fulfilling the standard design rules. There was therefore no possibility to exploit the impact of the reduction of $C_{P}$ through process optimization. In order to predict the impact of the combination of thin oxide SF with process optimizations reducing the sense node capacitance, the parameter $C_{P}$ of Equation (5) is replaced by the measurement results from [30]. The starting point corresponds to the result of $0.4\;e_{rms}^{-}$ obtained in [8] and corresponding to the case of a thin oxide SF-based pixel design with standard rules. Then, the values of $C_{P}$ based on [30] are used to predict the evolution of the input-referred noise for each additional technique used to reduce the the SN capacitance. The result is plotted in Figure 14. It shows the expected input-referred noise, at the optimal gate width and length, for each value of $C_{P}$. Figure 14 shows that the $0.3\;e_{rms}^{-}$ limit can be crossed if the thin oxide PMOS SF is combined with process optimizations reducing $C_{P}$ in the 180-nm process used in [8].

## 5. CIS Read Noise and Technology Downscaling

Since their first development, CIS pixels have always been designed with thick oxide transistors compatible with high voltages (3.3 V). The device parameters of thick oxide transistors do not follow the scaling rules as the thin oxide transistors. The impact of the technology downscaling on these devices is rather limited. Moreover, it appears that the oxide trap density of thick oxide transistors tends to increase with the technology downscaling, as shown in Figure 11.

**Figure 15:**
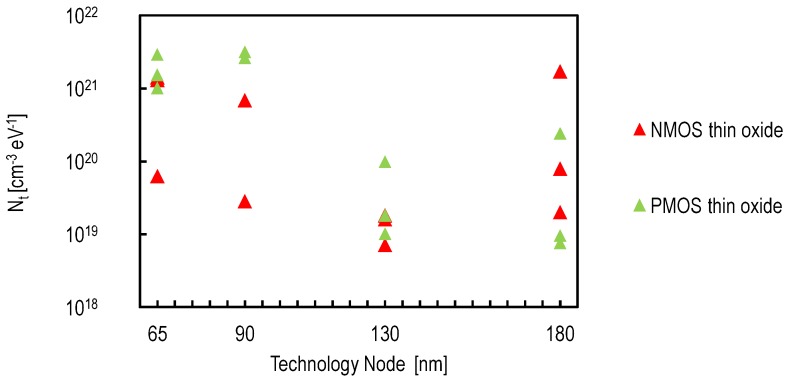
The evolution of the oxide trap density $N_{t}$, as a function of the technology node based on measurement results reported in design kits from different foundries.

It has been demonstrated in [8] that a thin oxide SF can be used together with a conventional PPD for a low noise performance. Thin oxide transistors, on the other hand, take full advantage of technology downscaling. Thus, it is interesting to investigate the impact of technology downscaling on the input-referred noise. The starting point for analyzing the impact of the technology downscaling on the input-referred noise of a readout chain based on a thin oxide SF is Equations (5) and (8). The conclusions can be made based on how the technology downscaling affects the different process and device parameters. Table 1 shows the scaling factor corresponding to the relevant device parameters [37]. The technology downscaling allows a higher oxide capacitance per unit area, a lower gate width and lower overlap and parasitic capacitances. Hence, the input-referred 1/f noise variance is supposed to decrease with $\kappa^{2}$, assuming that the oxide trap density $N_{t}$ remains constant with the technology downscale. The thermal noise is expected to decrease with $\kappa^{2}$ and, hence, would remain negligible. The International Technology Roadmap for Semiconductors (ITRS) expects the oxide trap density to decrease with the technology downscaling [22]. Figure 15 shows the $N_{t}$ values, for thin oxide transistors, reported in design kits of three foundries for different technology nodes. It shows that the oxide trap density follows the ITRS roadmap when downscaling from 180nm to 130nm. For more advanced technologies, the data are not conclusive and must be verified by measurements. The 1/f noise of NMOS transistors does not increase dramatically. On the contrary, PMOS transistors appear to show a higher $N_{t}$ for advanced technologies. In bulk CMOS, the buried channel conductance of the PMOS transistors is likely the reason for their lower 1/f noise. While deep submicron PMOS transistors are expected to behave as surface channel devices, which explains the fact that their 1/f noise becomes comparable to the one of NMOS transistors.

The measurement results presented in [8,38] explore indirectly the impact of the technology downscaling on the noise reduction. A pixel with a thin oxide SF transistor have been compared to a thick oxide SF based one. For the 180-nm process used in [8,38], the thick oxide transistor features an oxide capacitance per unit area of 5 fF/*μ*m${}^{2}$ compared to 9.55 fF/*μ*m${}^{2}$ for the thin oxide transistor. In addition, the minimum width determined by the design rules is 0.4μm for the thick oxide compared to 0.22μm for the thin oxide transistor. Consequently, using a thin oxide source follower transistor instead of a thick oxide has the same effect as a technology downscaling with a scaling factor of two. Based on this observation, the input-referred noise is expected to decrease by a factor of two, which matches the measurement results shown in Figure 13.

**Figure 16:**
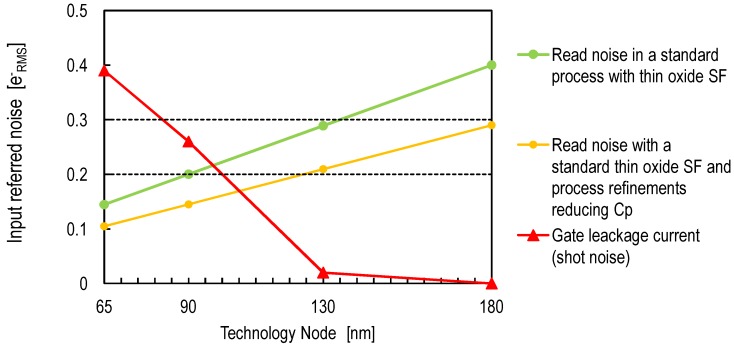
The expected evolution, with technology downscale, of the input-referred 1/f noise of CIS designed with standard CMOS process with thin oxide in-pixel SF.

Besides the read noise originating from the 1/f and thermal noise, the gate leakage current shot noise has been up to now neglected due to the extremely low levels of the leakage currents achieved in the used technology. It is important to investigate the evolution of this noise when using more advanced technologies. Indeed, the gate leakage current increases by several orders of magnitude when downscaling from 180-nm to 65-nm technologies [37]. Based on Equation (13), the shot noise associated with the gate leakage current is hence expected to increase significantly. In order to evaluate its impact, simulations have been performed with transistors having a minimum gate width and length from technologies between 180nm and 65nm. The corresponding leakage current shot noise RMS is given by the square root of the total number of electrons crossing the gate in a time interval of 10μs (enough to read two samples). The results are plotted in Figure 16. Figure 16 also shows how the input-referred noise is expected to decrease by only taking advantage of the technology downscaling based on Equation (5) and the assumption of constant oxide trap density for deep submicron technologies. The starting point corresponds to the input-referred noise obtained using a thin oxide SF in a 180-nm CMOS process [8]. It can be noticed that the $0.3\;e_{rms}^{-}$ limit can be crossed if a CIS process is developed with a technology node under 130nm and a thin oxide transistor is used as a SF. However, for technologies under 90nm, the gate leakage current appears to be a severe problem starting to dominate the total noise. Hence, the optimal technology node is between 130nm and 90nm, unless process improvements are applied to reduce the gate leakage current. Figure 16 shows also the impact on the technology node when the process refinements reducing the SN capacitance $C_{P}$ are applied. The noise levels for each technology node are obtained using Equation (5) and the scaling rules. The starting point corresponds to noise expected when combining the thin oxide PMOS SF with a $C_{P}$ of 0.25 fF, as shown in Figure 14. The latter shows that an input-referred read noise under $0.2\;e_{rms}^{-}$ could be possible with a technology node between 130nm and 90nm, a thin oxide SF and process level $C_{P}$ reduction.

RTS noise may also be a concern with the technology downscaling. In sate-of-the-art low noise CMOS image sensors, it may result in a dramatically high input referred-noise value of about several $e_{rms}^{-}$, but it is only present in a minority of pixels (the tail of the noise histogram). Therefore, RTS noise was not accounted for in this work, including in the extrapolation towards downscaled technologies, because we limited the latter to 65 nm, where leakage is much more an issue. A further investigation of input-referred noise for such 4T pixels in deep submicron technology would definitely require one to account for RTS noise. Unfortunately, RTS noise is not modeled in the most common simulators, and the complexity of this phenomena still impedes an analytically- or empirically-precise expression of its occurrence.

## 6. Conclusions

The capability of performing photo-electron counting, with an accuracy higher than 90%, using conventional CIS readout chains requires a total read noise level below $0.3\;e_{rms}^{-}$. This read noise is mainly composed of the 1/f noise originating from the in-pixel SF, the thermal noise originating from the pixel- and column-level saturated transistors and the shot noise associated with the leakage current at the level of the SN. The latter is negligible in the technology nodes used currently (above 100nm).

The thermal noise can be drastically reduced, to extremely low levels, by combining column-level gain, bandwidth control and CMS. The 1/f noise becomes then the dominant noise source. The reduction of the 1/f noise can involve process-, device- and circuit-level optimizations. The process-level refinements include the sense node total capacitance and the SF $K_{F}$ parameter reduction. At the device level, the input-referred noise can be reduced by using an in-pixel SF with a higher oxide capacitance per unit area, a minimum gate width and an optimal gate length. The implementation of an in-pixel thin oxide PMOS SF-instead of a thick oxide NMOS presents a practical example of how this device level optimization can be performed in a standard process. At the circuit level, the 1/f noise can be slightly further reduced using the CMS.

Based on measurement results reported in recent works and the analytical expressions of the input-referred noise, the combination of a standard thin oxide PMOS SF with the process refinement reducing the SN capacitance is expected to decrease the total read noise of a conventional CIS below $0.3\;e_{rms}^{-}$.

The input-referred thermal and 1/f noise are expected to decrease with the technology downscaling to levels below $0.2\;e_{rms}^{-}$. For technologies below 90nm, the SF gate oxide leakage current is expected to increase dramatically. Therefore, unless the leakage current is reduced by some other means at the process level, the optimum technology node ranges between 90nm and 130nm.

## Figures and Tables

**Table 1:** Impact of technology downscaling on the parameters of the input-referred 1/f noise in Equation (5).

Parameter	Scaling Factor
*W*	$\frac{1}{\kappa }$
*L*	$\frac{1}{\kappa }$
$C_{ox}$	*κ*
$C_{P}$	$\frac{1}{\kappa }$
$C_{e}\cdot W$	$\frac{1}{\kappa }$
